# Cannabidiol potentiates olaparib-induced cytotoxicity through cell cycle arrest and DNA damage modulation in breast cancer cells

**DOI:** 10.1007/s11010-026-05550-w

**Published:** 2026-04-28

**Authors:** Cansın Deniz, Büşra Yüksel, Emre Cebeci, Fikrettin Şahin

**Affiliations:** Faculty of Engineering and Natural Sciences, Genetics and Bioengineering Department, Yeditepe University, 34755 Kayışdağı, Istanbul Turkey

**Keywords:** TNBC, Breast Cancer, Olaparib, CBD

## Abstract

Triple-negative breast cancer (TNBC) remains a major clinical challenge due to its aggressive nature and limited treatment options, while therapeutic resistance in estrogen receptor-positive (ER+) breast cancer continues to limit treatment efficacy. Although olaparib is primarily effective in BRCA-mutated cancers, its activity in BRCA-wild type (BRCA-wt) tumors is limited. Therefore, this study aimed to investigate whether cannabidiol (CBD) can enhance the response of BRCA-wt breast cancer cells to PARP inhibition. The effects of olaparib (OLAP) and CBD, alone and in combination, were evaluated in MDA-MB-231 (TNBC) and MCF-7 (ER+) cell lines using comprehensive two-dimensional (2D) mechanistic analyses and three-dimensional (3D) spheroid models, including HCC-70 cells to extend TNBC validation. The results demonstrate that combined OLAP and CBD treatment enhanced cytotoxic effects compared to single treatments, with more pronounced responses observed in 3D spheroid models, particularly in TNBC models. Flow cytometry and caspase 3/7 assays indicated increased apoptosis and G2​/M phase arrest following combination treatment. Gene expression analysis revealed downregulation of key DNA damage response and cell cycle-related genes (*ATM*,* ATR*,* BRCA1/2*,* RAD51*, and *CDK1/2/4/6*), supporting a role for cell cycle arrest and DNA damage modulation in mediating these effects. Functional assays showed reduced colony formation and migratory capacity, although these effects may reflect both cytotoxic and cytostatic responses under the selected experimental conditions. Overall, these findings suggest that CBD may enhance the efficacy of olaparib in BRCA-wt breast cancer models and highlight its potential as a combinational therapeutic strategy in breast cancer treatment.

**Supplementary Information:**

The online version contains supplementary material available at 10.1007/s11010-026-05550-w.

## Introduction

Cancer is a complex disease characterized by diverse subtypes, multifactorial etiology, and profound physiological alterations that lead to malignant tumor formation [1]. Among women, breast cancer is the most commonly diagnosed malignancy. Although it predominantly affects women over the age of 50, breast cancer can also occur in younger individuals and, less frequently, in men [2]. When detected at an early stage, breast cancer is often curable; however, invasive and advanced forms remain challenging to treat. Disease staging is determined based on tumor size, lymph node involvement, and the presence of distant metastasis [3, 4]. While early-stage cancers generally respond well to treatment, advanced-stage disease is associated with an increased risk of metastasis and requires more complex therapeutic strategies [5, 6].

Despite advances in targeted therapies, breast cancer treatment remains a significant clinical challenge due to drug resistance, tumor heterogeneity, and the adverse side effects associated with conventional therapies [7]. In this context, natural compounds are increasingly investigated as alternative or complementary therapeutic options [8]. These bioactive substances, derived primarily from plants or other natural sources, have demonstrated anticancer properties, including reduced toxicity, inhibition of metastasis, and induction of apoptosis [9]. Consequently, the integration of natural compounds into cancer treatment strategies has gained growing interest in both preclinical and clinical research settings [10].

Cannabinoids are bioactive compounds originating from the cannabis plant and are classified into phytocannabinoids, endocannabinoids, and synthetic cannabinoids [11, 12]. Among these, Δ9-tetrahydrocannabinol (THC) and cannabidiol (CBD) are the most extensively studied. Although only a limited number of cannabinoids, such as dronabinol and cannabidiol, have received FDA approval, research exploring their therapeutic potential, particularly in oncology, has expanded substantially in recent years [13]. Cannabinoids have been reported to enhance the effects of conventional cancer treatments, including chemotherapy and radiotherapy, thereby improving therapeutic efficacy while potentially reducing treatment-related toxicity [14, 15].

CBD, in particular, has shown promising antitumor activity in triple-negative breast cancer (TNBC) by inducing apoptosis, autophagy, and oxidative stress through modulation of signaling pathways such as AKT/mTOR [16]. In combination-based approaches, CBD has been reported to enhance treatment responses and mitigate adverse effects; however, further clinical investigation is required to establish its role in routine cancer therapy [17, 18]. Recent studies have also demonstrated that cannabinoids, especially THC and CBD, can significantly reduce the motility and invasive capacity of breast cancer cells, processes that are critical for metastatic progression [19]. In vitro findings suggest that these phytocannabinoids may suppress tumor progression by impairing cancer cell migration, although the majority of available evidence remains preclinical [20].

Olaparib (Lynparza) is a poly(ADP-ribose) polymerase (PARP) inhibitor that disrupts DNA repair mechanisms in cancer cells and has demonstrated particular efficacy in BRCA1/2-mutated tumors [21]. Initially approved in 2014, olaparib is currently used in the treatment of several malignancies, including breast, ovarian, pancreatic, and prostate cancers [22]. It may be administered as monotherapy in BRCA-mutated cases or combined with surgery, radiotherapy, chemotherapy, or immunotherapy in advanced disease settings. However, the efficacy of PARP inhibitors such as Olaparib in BRCA-wild type (BRCA-wt) breast cancers remains limited, highlighting the need for combinational strategies to enhance therapeutic response in these models [23, 24]. Ongoing research continues to explore both its standalone efficacy and its potential use in combination with natural compounds to improve therapeutic outcomes [25]. While current findings are promising, further studies are required to establish its full therapeutic potential.

Combination strategies that integrate targeted therapies with bioactive natural compounds have been increasingly investigated to enhance antitumor efficacy and potentially improve therapeutic outcomes [26, 27]. Two-dimensional (2D) cell culture models provide a simplified and reproducible system for initial drug screening; however, they fail to fully recapitulate the complexity of the tumor microenvironment. In contrast, three-dimensional (3D) culture models better mimic in vivo tumor architecture, cell–cell interactions, and drug penetration gradients, resulting in more physiologically relevant treatment responses [27].

In this study, the antitumor effects of Cannabidiol (CBD) and Olaparib (OLAP), alone and in combination, were investigated in triple-negative (TNBC) and estrogen receptor-positive (ER+) breast cancer models. To provide mechanistic insight, 2D analyses of cell cycle regulation, apoptosis, and DNA damage signaling were integrated with 3D spheroid-based validation. The study specifically aimed to determine whether CBD enhances the cytotoxic effects of Olaparib through modulation of key survival pathways, particularly in BRCA-wild type breast cancer models. This approach enables a more comprehensive evaluation of the therapeutic potential of this combination in aggressive breast cancer subtypes.

## Materials & methods

### Cell lines & cell culture conditions

MDA-MB-231 (CRM-HTB-26), MCF-7 (HTB-22), and HCC-70 (CRL-2315) human breast cancer cell lines were obtained from the American Type Culture Collection (ATCC, Rockville, MD, USA). MDA-MB-231 and MCF-7 cells were cultured in Dulbecco’s Modified Eagle’s Medium (DMEM; #41966-029, Gibco, Invitrogen, UK), whereas HCC-70 cells were maintained in RPMI-1640 medium (#30–2001, Gibco, Invitrogen, UK). All media were supplemented with 10% fetal bovine serum (FBS; #10500-064, Gibco, Invitrogen, UK) and 1% penicillin–streptomycin–amphotericin B solution (PSA; Gibco, Invitrogen, UK). Cells were maintained in a humidified incubator at 37 °C with 5% CO₂.

### Olaparib and CBD preparation

Olaparib (Olap; LC Laboratories, Cat. No. O9201) and cannabidiol (CBD; TCl Chemical, Cat.No. TCI-T2744) were dissolved in dimethyl sulfoxide (DMSO; Sigma-Aldrich, USA; Cat. No. D2650) to prepare stock solutions at concentrations of 2000 µM and 1000 µM, respectively. Stock solutions were sterilized by filtration through a 0.22 μm syringe filter and subsequently diluted in DMEM to obtain the desired working concentrations for each experiment.

### Cytotoxicity assay

The effects of olaparib and cannabidiol (CBD) on cell viability were evaluated in MDA-MB-231 and MCF-7 breast cancer cell lines. Stock solutions of olaparib and CBD were prepared in dimethyl sulfoxide (DMSO) and diluted in culture medium prior to treatment.

MDA-MB-231 and MCF-7 cells were seeded in 96-well plates at densities of 5,000 and 3,000 cells per well, respectively. After 24 h of incubation, cells were treated with olaparib (31.25–2000 µM) and CBD (6.25–200 µM) using two-fold serial dilutions.

For combination treatments, CBD was applied at fixed concentrations (50 µM for MDA-MB-231 and 25 µM for MCF-7), while olaparib concentrations were varied starting from 2 µM.

Following treatment, cells were incubated for 24, 48, and 72 h. Cell viability was assessed using the MTS assay (3-(4,5-dimethylthiazol-2-yl)−5-(3-carboxymethoxyphenyl)−2-(4-sulfophenyl)−2 H-tetrazolium) (CellTiter 96 AQueous One Solution, #G3582; Promega, Southampton, UK) according to the manufacturer’s instructions. Briefly, treatment-containing medium was removed, and MTS solution (prepared in PBS containing 10% MTS and 4.5 g/L D-glucose) was added, followed by incubation at 37 °C for 60 min. Absorbance was measured at 490 nm using a microplate reader (BioTek, Winooski, VT, USA). IC_50_ values were calculated using GraphPad Prism software.

### Combination index (CI) analysis

Drug interactions between olaparib and cannabidiol (CBD) were evaluated using the Chou–Talalay median-effect method. Cells were treated with olaparib alone, CBD alone, or their combination, and cell viability was assessed using the MTS assay. IC_50_ values were calculated using GraphPad Prism software.

The combination index (CI) was calculated using the following equation:

$${\mathrm{CI}} = \left( {{\mathrm{D}}_{{\mathrm{1}}} /{\mathrm{Dx}}_{1} } \right) + \left( {{\mathrm{D}}_{{\mathrm{2}}} /{\mathrm{Dx}}_{2} } \right)$$

where Dx_1_ and Dx_2_ represent the IC_50_ values of olaparib and CBD alone, respectively, and D₁ and D₂ represent the concentrations of each agent in combination required to achieve the same level of inhibition (50% cell viability).

CI values < 1 indicate synergistic effects, CI = 1 indicates an additive effect, and CI > 1 indicates antagonistic interactions.

### Annexin V/propidium iodide (PI) assay

The concentrations of olaparib and cannabidiol used for apoptosis analysis by the Annexin V/PI assay were determined based on IC_50_ values obtained from the MTS cytotoxicity assay. The MDA-MB-231 cell line was seeded into T-25 flasks at 200,000 cells per flask for treatment and negative control groups. After 24 h, the culture medium was discarded, and cells were treated with 125 µM olaparib and 50 µM cannabidiol or their combination. Similarly, MCF-7 cell line was seeded into T-25 flasks at a density of 120,000 cells per flask for treatment and negative control groups. After 24 h, the medium was aspirated, and cells were treated with 500 µM olaparib, 25 µM cannabidiol or their combination. For negative control groups, the medium was replaced with fresh culture medium. After 72 h of treatment, apoptosis was evaluated using the Annexin V/PI apoptosis detection assay according to the manufacturer’s instructions (#sc-4252AK, Santa Cruz Biotechnology, USA). Cells were harvested, washed with ice-cold PBS, resuspended in Annexin V binding buffer and stained with Annexin V and/or propidium iodide. Samples were analyzed in four groups: Annexin V-positive, propidium iodide-positive, Annexin V/PI double-positive, and unstained negative control. Data acquisition and analysis were performed using a FACSCalibur flow cytometer (BD Biosciences).

### Caspase 3/7 activity assay

Caspase activity was evaluated using Caspase-Glo^®^ 3/7 assay systems (Promega, Madison, WI, USA) according to the manufacturer’s instructions. Cells were seeded in white 96-well plates and allowed to attach for 24 h. Subsequently, cells were treated with the indicated compounds or their combinations for 72 h. Following treatment, Caspase-Glo reagents were added to each well and incubated for 60 min at room temperature. Luminescence was measured using a microplate luminometer (Thermo Scientific Varioskan Lux), and caspase activity was quantified relative to the untreated control group.

### Cell cycle analysis

Cell cycle analysis was performed using the same treatment conditions and concentrations applied in the Annexin V/PI apoptosis assay. Cells were seeded at the same densities as those used for the apoptosis experiments and incubated at 37 °C in a humidified atmosphere containing 5% CO₂. After 24 h, cells were treated with olaparib, cannabidiol, or their combination at the same concentrations described above. Following 72 h of treatment, cells were harvested, washed with PBS, and fixed in 70% ice-cold ethanol at − 20 °C for at least 2 h. Fixed cells were then permeabilized with 0.1% Triton X-100 and incubated with RNase A (20 µg/mL) for 30 min at room temperature. Subsequently, cells were stained with propidium iodide (PI), and cell cycle distribution was analyzed within 15 min using flow cytometry equipped with a 488 nm laser.

### Real-time PCR

Total RNA was isolated by using an RNA isolation kit (#740955.250, Macherey-NAGEL, Düren, Germany) according to the manufacturer’s instructions. The concentration and purity of the isolated RNA were determined spectrophotometrically. Subsequently, complementary DNA (cDNA) was synthesized from total RNA using the QuantiTect Reverse Transcription Kit (#205313, QIAGEN, Hilden, Germany). Quantitative real-time PCR (RT-qPCR) was performed using SYBR Green chemistry (#4309155, Thermo Fisher Scientific, Waltham, MA, USA) on an iCycler real-time PCR detection system (Bio-Rad, Hercules, CA, USA). All reactions were carried out in technical triplicates. Gene expression levels were normalized to the housekeeping gene RPL30 (ribosomal protein L30). The primer sequences of the target genes analyzed in this study are listed in Table 1.

### Colony formation assay

MCF-7 cells were seeded in 6-well plates at a density of 1,000 cells per well while MDA-MB-231 cells were seeded at 250 cells per well. Colonies were allowed to form for 3 days in MCF-7 cells and 10 days in MDA-MB-231 cells respectively, due to differences in proliferation rates and colony-forming capacity between the cell lines. Following colony formation, MCF-7 cells were treated with olaparib 500 µM, cannabidiol 25 µM, or their combination whereas MDA-MB-231 cells were treated with olaparib 125 µM, cannabidiol 50 µM, or their combination. After 3 days of treatment, the culture medium was removed, and colonies were stained with 800 µL of 0.5% methylene blue solution for 30 min. Wells were then gently washed with PBS, and images of the stained colonies were captured using a plate imaging system (Bio-Rad, USA).

**Table 1:** Gene pairs with forward and reverse primers were employed

Gene	Side	Gene sequence (from 5′ to 3′)
*LC3B*	*FORWARD*	AGCAGCATCCAACCAAAATC
	*REVERSE*	CTGTGTCCGTTCACCAACAG
TP53	*FORWARD*	GCCCAACAACACCAGCTCCT
	*REVERSE*	CCTGGGCATCCTTGAGTTCC
*ATM*	*FORWARD*	TGTTCCAGGACACGAAGGGAGA
	*REVERSE*	CAGGGTTCTCAGCACTATGGGA
*ATR*	*FORWARD*	GGAGATTTCCTGAGCATGTTCGG
	*REVERSE*	GGCTTCTTTACTCCAGACCAATC
*BRCA 1*	*FORWARD*	CAGTGTCAGGTTGTGCTTGC
	*REVERSE*	AGTAGTGGCTGTGGGGTTTC
*BRCA 2*	*FORWARD*	GGTCCTGGAATTCGTCTGGA
	*REVERSE*	CCTGTGTTGGAAGGCTCTGA
BIRC5	*FORWARD*	TCTTCACCGCTTTGCTTTC
	*REVERSE*	CGCACTTTCTCCGCAGTTTC
*PARP 1*	*FORWARD*	CAGGAGAAGGCTGTTGGAGA
	*REVERSE*	GTGAGGTGGTGTCAGGTTCC
BECN 1	*FORWARD*	ATGGAAGGGTCTAAGACGTCC
	*REVERSE*	CTGTTGGCACTTTCTGTGGG
*PCNA*	*FORWARD*	CAAGTAATGTCGATAAAGAGGAGG
	*REVERSE*	GTGTCACCGTTGAAGAGAGTGG
*CDK 1*	*FORWARD*	CACTTGGCTTCAAAGCTGGCTC
	*REVERSE*	ATGGGTATGGTAGATCCCGGC
*CDK 2*	*FORWARD*	CTGGACACGCTGCTGGATG
	*REVERSE*	ATGCCAGTGAGAGCAGAGGC
*CDK 4*	*FORWARD*	GTCTATGGTCGGGCCCTCTG
	*REVERSE*	CAGATCAAGGGAGACCCTCACG
*CDK 6*	*FORWARD*	GTCTGATTACCTGCTCCGCGA
	*REVERSE*	TCCAGAATCATTGCACCTGAGGG
*KI 67*	*FORWARD*	GAAAGAGTGGCAACCTGCCTTC
	*REVERSE*	GCACCAAGTTTTACTACATCTGCC
*ATG 5*	*FORWARD*	GGCCATCAATCGGAAACTC
	*REVERSE*	AGGTCTTTCAGTCGTTGTCT
*RAD 51*	*FORWARD*	ATGGCAATGCAGATGCAGC
	*REVERSE*	TCAGTCTTTGGCATCTCCGT
*RPL 30*	*FORWARD*	GGCTGCAAAGGAGATCAAGG
	*REVERSE*	TCCAGCTCAGCAAAGGACAC

The Primer-BLAST online application from The National Centre for Biotechnology was used to produce sequences written from 5′ end to 3′ end primers

### Spheroid formation

For the MCF-7 cell line, 96-well plates were coated with 1% agarose to prevent cell attachment. Subsequently, 1,000 MCF-7 cells were seeded per well. The cells were incubated for 7 days at 37 °C and 5% CO₂, allowing spheroid formation.

For the HCC-70 cell line, spheroids were generated using the hanging drop method. Briefly, 125,000 cells were suspended in 1 mL of medium, and 10 µL drops were placed on the inner surface of a petri dish lid. The lid was then inverted over a dish containing PBS to maintain humidity, and the cells were left suspended. On the fourth day, spheroids formed in the drops were transferred into 96-well plates pre-coated with 1% agarose for further experiments. Images of the spheroids were obtained using a Zeiss inverted microscope with a 10X objective.

### Spheroid cell viability assay

After spheroid formation, MCF-7 spheroids were treated with olaparib at concentrations ranging from 2000 µM to 15.625 µM, cannabidiol at concentrations ranging from 1000 µM to 7.81 µM, and their combination (cannabidiol fixed at 7.81 µM and olaparib at concentrations ranging from 500 µM to 15.125 µM). HCC-70 spheroids were treated with olaparib at concentrations ranging from 1000 µM to 31.25 µM, cannabidiol at concentrations ranging from 500 µM to 15.625 µM, and their combination (cannabidiol fixed at 15.625 µM and olaparib at concentrations ranging from 1000 µM to 15.625 µM). Treatments were applied for 72 h. Following treatment, cell viability was assessed using the CellTiter-Glo^®^ 3D Cell Viability Assay (Promega, Southampton, UK) according to the manufacturer’s instructions. Luminescence was measured using a plate reader, and results were used to determine treatment efficacy. Images of the spheroids were obtained using a Zeiss inverted microscope with a 10X objective.

### Statistical analysis

All data are shown as the means ± standard errors. The statistical analysis of the results was performed with an two-way ANOVA, and graphs were drawn using GraphPad Prism 5 software. Statistical significance was determined at ns: non-significant, *: *p* < 0.05, **: *p* < 0.01, ***: *p* < 0.001, ****: *p* < 0.0001.

## Results

### The effect of olaparib (OLAP) and cannabidiol (CBD) on cell survival, both individually and in combination

**Fig. 1:**
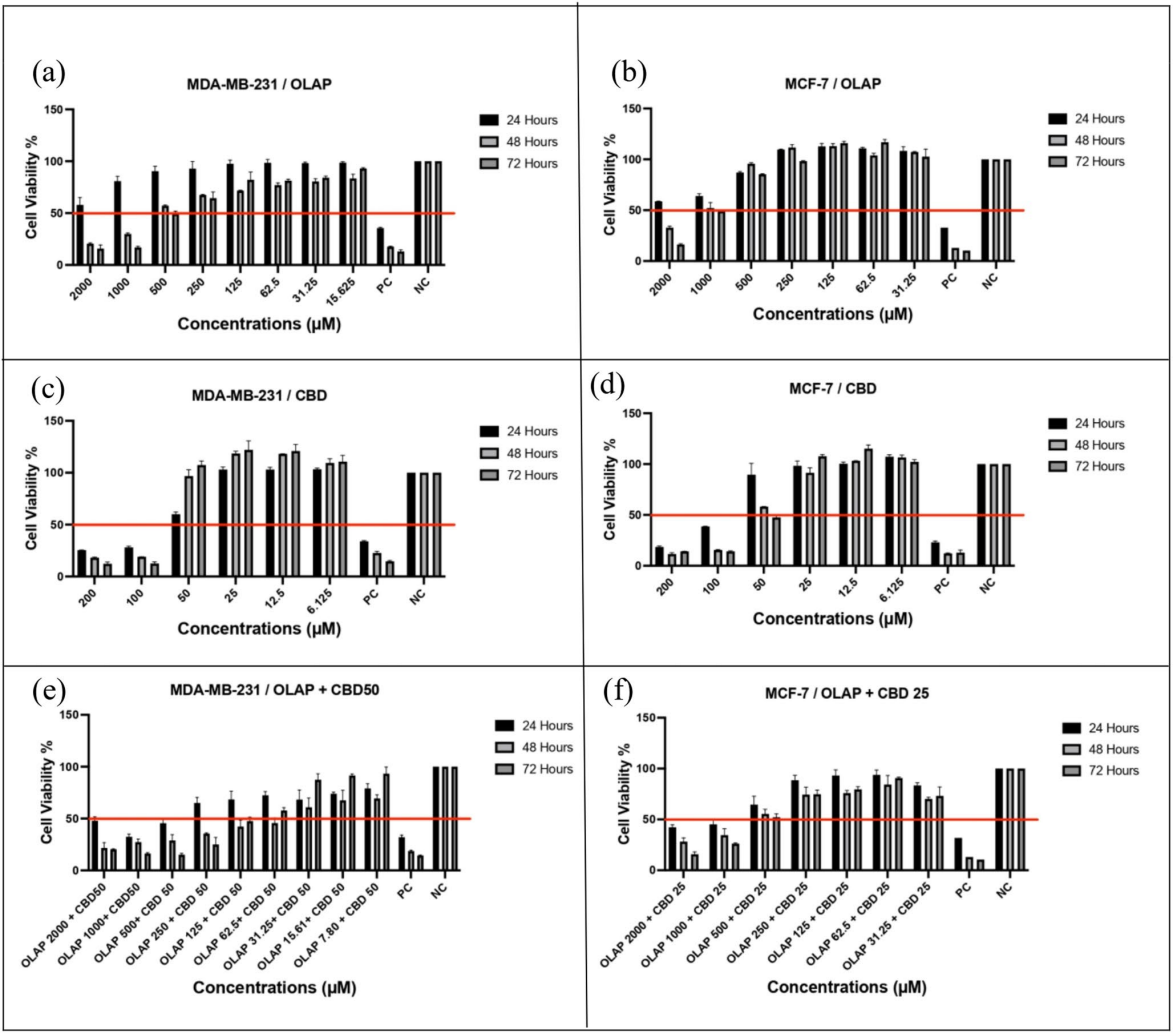
Comparison of cell viability (%) in MDA-MB-231 and MCF-7 cell lines at 24, 48, and 72 h. Graphs represent cells treated with **a**, **b** OLAP, **c**, **d** CBD, and **e**, **f** OLAP + CBD combination. Left panels **a**, **c**, **e** show results for MDA-MB-231, while right panels **b**, **d**, **f** show results for MCF-7. Data are expressed as mean ± SD (*n* = 3). NC stands for negative control (untreated cells), while PC represents positive control (cells treated with a cytotoxic (DMSO) agent to induce cell death)

One of the most widely used in vitro methods for evaluating the anticancer potential of natural compounds and synthetic agents is the MTS cell proliferation assay. This colorimetric assay is reliable, sensitive, and applicable to a wide range of cell types [28]. The effects of olaparib (OLAP) and cannabidiol (CBD), both individually and in combination, on the viability of MCF-7 and MDA-MB-231 cells were assessed using an MTS-based cytotoxicity assay over 72 h (Fig. 1). A range of concentrations was tested for each cell line to determine IC_50_ values. For MDA-MB-231 cells, OLAP concentrations ranged from 2000 to 15.625 µM, whereas for MCF-7 cells, concentrations ranged from 2000 to 31.25 µM. CBD concentrations ranged from 200 to 6.25 µM for both cell lines.

IC_50_ values (​​Half-maximum inhibitory concentration is defined as the concentration that results in 50% cell viability [29]), ​​are shown in Table 2.

IC_50_ values, defined as the concentration resulting in 50% cell viability [29], are presented in Table 2. Single-agent treatments with OLAP and CBD reduced cell viability in a dose-dependent manner. The IC_50_ values for OLAP were 907.5 µM in MCF-7 cells (Fig. S1e) and 493.2 µM in MDA-MB-231 cells (Fig. S1b), whereas for CBD, the IC_50_ values were 48.8 µM in MCF-7 cells (Fig. S1d) and 62.5 µM in MDA-MB-231 cells (Fig. S1a).

For combination treatments, CBD was applied at fixed concentrations (50 µM for MDA-MB-231 and 25 µM for MCF-7), while olaparib concentrations were varied. In MDA-MB-231 cells, 78.93 µM olaparib in combination with 50 µM CBD resulted in 50% cell viability (Fig. S1c). In MCF-7 cells, 603.6 µM olaparib combined with 25 µM CBD achieved a similar effect (Fig. S1f). Also, CI analysis based on the Chou–Talalay method revealed a synergistic interaction in MDA-MB-231 cells (CI ≈ 0.96), whereas an antagonistic effect was observed in MCF-7 cells (CI ≈ 1.18).

Overall, these findings indicate that cannabidiol modulates the cytotoxic response to olaparib in a cell line–dependent manner under fixed-dose conditions. While both OLAP and CBD reduced cell viability as single agents, their combination resulted in a modest synergistic effect in MDA-MB-231 cells, whereas an antagonistic interaction was observed in MCF-7 cells (CI > 1). These findings likely reflect intrinsic molecular differences between the cell lines and suggest that the therapeutic response to this combination is highly dependent on cellular context.

**Table 2:** IC_50_ values of treatment for MDA-MB-231 and MCF-7 cell

Treatment	Combination dose of CBD (µM)	IC_50_ values of MDA-MB-231	IC_50_ values of MCF-7
Olaparib	–	493.2 µM	907.5 µM
Cannabidiol	–	62.5 µM	48.8 µM
Olaparib + CBD	50	78.93 µM	–
Olaparib + CBD	25	–	603.6 µM

### The effect of olaparib (OLAP) and cannabidiol (CBD) combination treatment on apoptosis in breast cancer cell lines

Apoptosis, a key mechanism of programmed cell death, plays a critical role in tumor cell susceptibility to chemotherapeutic agents [30]. The effects of OLAP, CBD, and their combination on apoptosis were evaluated in MDA-MB-231 and MCF-7 cells using Annexin-V staining and flow cytometry, supported by caspase activity assays.

**Fig. 2:**
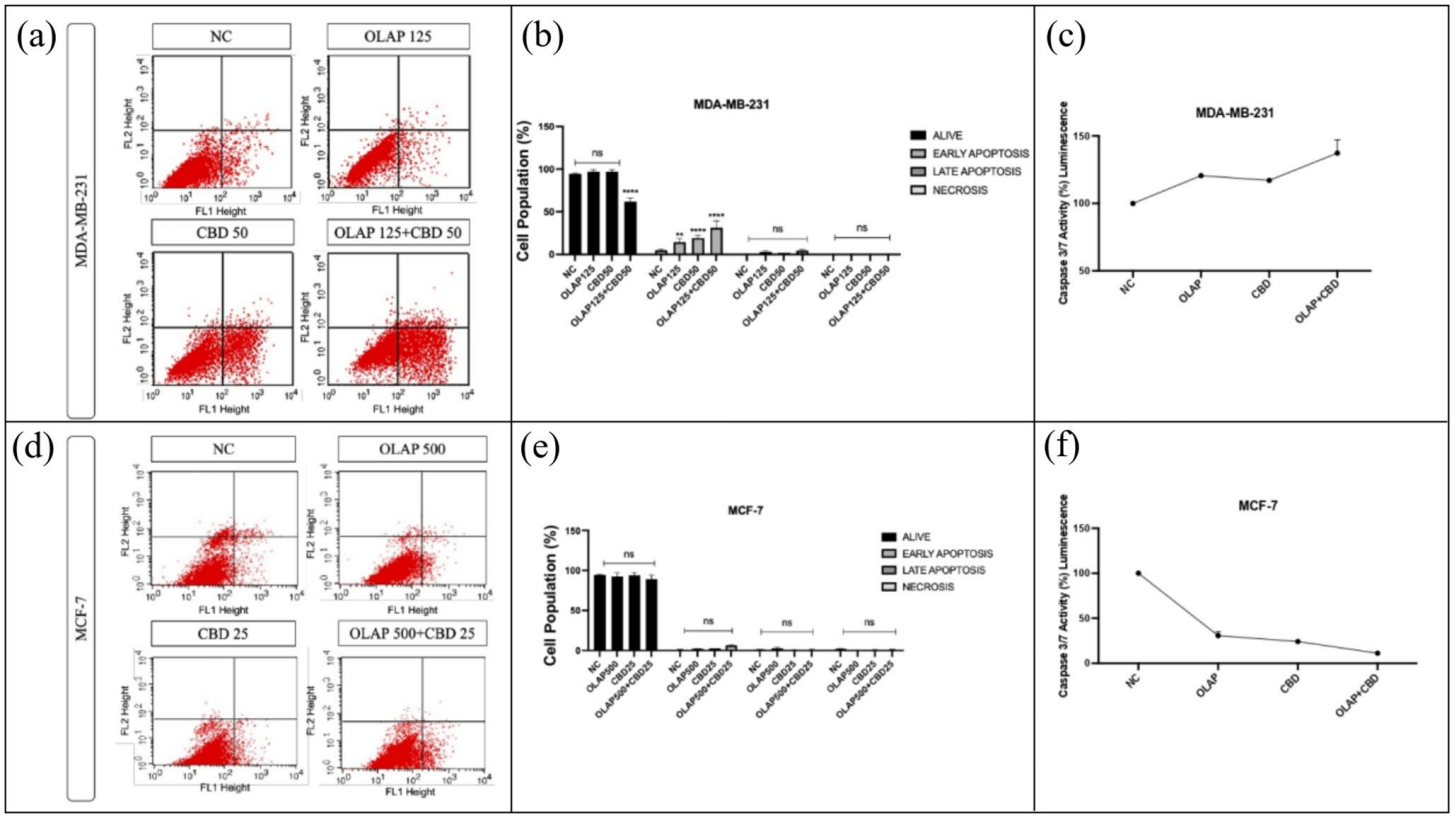
Induction of apoptosis and caspase activation in MDA-MB-231 and MCF-7 cells after 72 h of treatment. The upper row **a**–**c** corresponds to MDA-MB-231 cells, and the lower row **d**–**f** corresponds to MCF-7 cells. **a**, **d** Representative Annexin V-FITC/PI flow cytometry dot plots showing the distribution of cell populations (viable, early/late apoptotic, and necrotic). **b**, **e** Quantitative analysis of the percentage of apoptotic cell populations. **c**, **f** Relative Caspase 3/7 activity compared to the untreated control group. The abbreviation NC stands for negative control (untreated control cells). All data are presented as mean ± SD of three independent experiments (*n* = 3). Statistical significance is indicated as ns (non-significant), *p < 0.05, **p < 0.01, ***p < 0.001, ****p < 0.0001. Data are presented as mean ± SD (*n* = 3)

In MDA-MB-231 cells, combination treatment increased early apoptosis compared to single-agent treatments and the negative control, with 31.49% of cells in the early apoptotic population (Fig. 2a and b). Single-agent treatments also induced early apoptosis, but to a lesser extent. Total apoptotic fractions (early + late apoptosis) were also increased following combination treatment. Flow cytometry analysis further revealed the distribution of viable, early apoptotic, late apoptotic, and necrotic cells, as previously decribed for Annexin V-based apoptosis assays [31]. In contrast, MCF-7 cells showed no significant increase in apoptosis or necrosis after any treatment (Fig. 2d and e), with only 6.43% of cells in the early apoptotic population.

Caspase-3/7 activity, markers of executioner caspases in apoptosis, increased in MDA-MB-231 cells following combination treatment (Fig. 2c), confirming induction of apoptosis. In MDA-MB-231 cells, caspase-3/7 activity increased following combination treatment, reaching approximately 137% compared to the negative control (Fig. 2c). In contrast, MCF-7 cells exhibited a marked reduction in caspase-3/7 activity, decreasing from 100% in the control group to approximately 11% after treatment (Fig. 2f). MCF-7 cells are known to lack functional caspase-3 expression due to a gene deletion, which may contribute to reduced caspase 3/7 activity and suggests that apoptosis may occur through caspase-independent mechanisms [32]. This may also explain the limited apoptotic response observed with olaparib treatment alone in MCF-7 cells. In addition, the BRCA-wild type status of MCF-7 cells may further contribute to reduced sensitivity to PARP inhibition, as intact homologous recombination repair mechanisms can limit the effectiveness of olaparib.

### The effect of olaparib (OLAP) and cannabidiol (CBD) combination treatment on cell cycle in breast cancer cell lines

The cell cycle is a tightly regulated process governed by cyclin-dependent kinases (CDKs) and their inhibitors, and disruption of this regulation is a key feature of cancer development [33, 34]. To investigate whether OLAP, CBD, and their combination affect cell cycle regulation, flow cytometry-based cell cycle analysis and qPCR were performed in MDA-MB-231 and MCF-7 breast cancer cell lines.

**Fig. 3:**
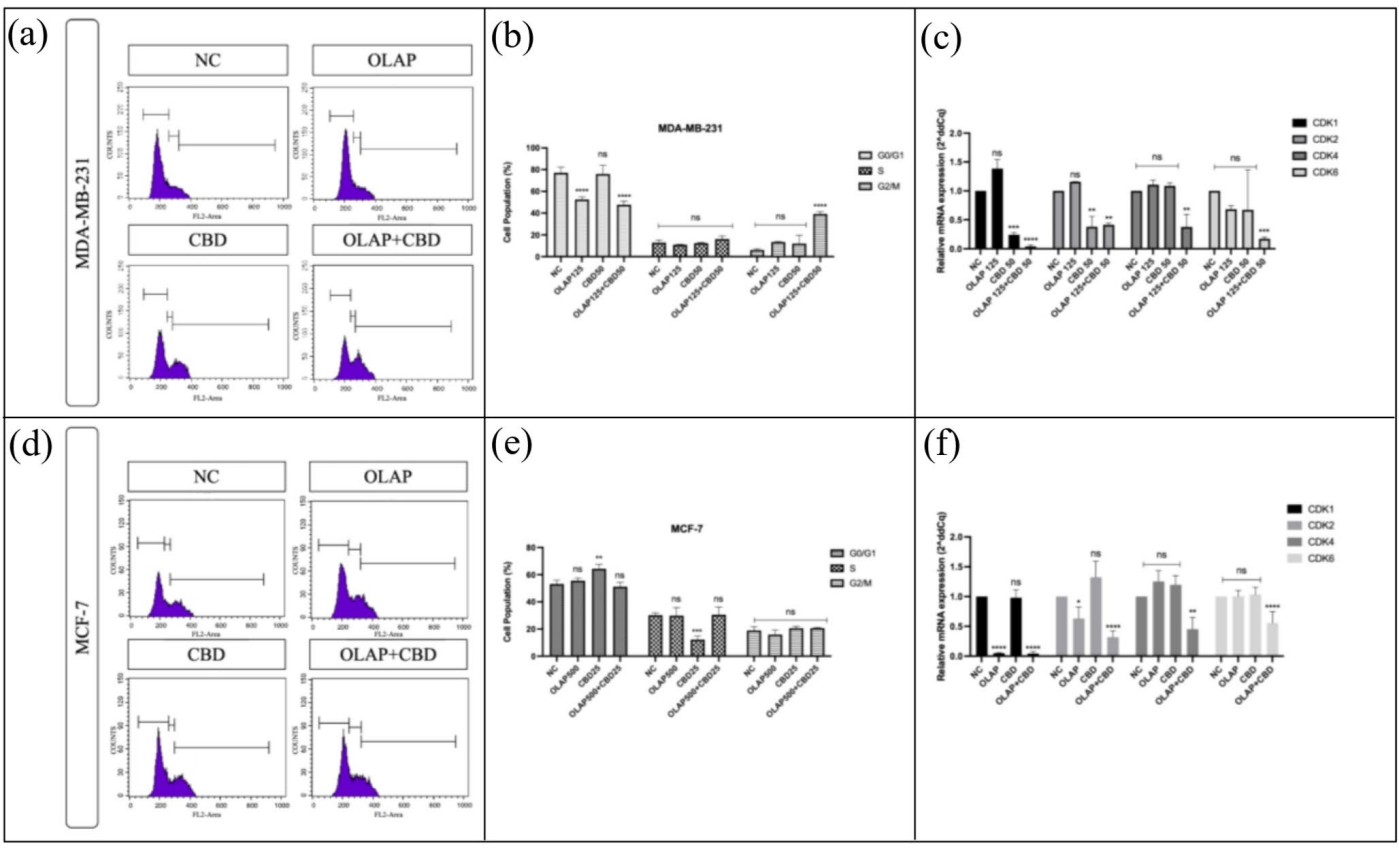
Cell cycle distribution and mRNA expression of cell cycle-related genes in MDA-MB-231 and MCF-7 cells after 72 h. Upper panels **a**–**c** represent MDA-MB-231 cells and lower panels **d**–**f** represent MCF-7 cells. **a**, **d **Representative flow cytometry histograms showing cell cycle phase distribution (G0/G1, S, and G2/M). **b**, **e **Quantitative analysis of the percentage of cells in each cell cycle phase. **c**, **f** Relative mRNA expression levels of CDK1, CDK2, CDK4, and CDK6 genes determined by qPCR. The abbreviation NC stands for negative control (untreated control cells). All data are presented as mean ± SD of three independent experiments (*n* = 3). Statistical significance is indicated as ns (non-significant), *p < 0.05, **p < 0.01, ***p < 0.001, ****p < 0.0001

Flow cytometric analysis revealed a shift in cell cycle distribution in MDA-MB-231 cells following combination treatment. A reduction in the G0/G1 population was observed, accompanied by an accumulation of cells in the G2/M phase, indicating a potential G2/M phase cell cycle arrest (Fig. 3a and b). In the combination treatment group, CDK1 expression decreased markedly from 1.00 in the negative control to approximately 0.04. Similarly, CDK2, CDK4, and CDK6 expression levels were reduced to approximately 0.41, 0.37, and 0.17, respectively (Fig. 3c).

In contrast, flow cytometry analysis of MCF-7 cells showed no significant accumulation in any specific phase of the cell cycle following treatment when compared with the negative control, indicating the absence of a pronounced cell cycle arrest (Fig. 3d and e). Consistent with these findings, qPCR analysis demonstrated a moderate downregulation of CDK gene expression in the combination treatment group. CDK1 expression decreased to approximately 0.04, while CDK2, CDK4, and CDK6 expression levels were reduced to 0.30, 0.45, and 0.55, respectively (Fig. 3f). These results suggest that MCF-7 cells are less responsive to OLAP and CBD-induced cell cycle perturbations under the tested conditions.

### The effect of OLAP and CBD combination therapy on DNA damage mechanism and cell proliferation in breast cancer cell lines

DNA damage response is a key mechanism for maintaining genomic integrity [34, 35]. To evaluate the effect of the combination treatment on this pathway, the expression levels of ATM, ATR, PARP1, RAD51, BRCA1, and BRCA2 were analyzed by qPCR. Cell proliferation is a hallmark of cancer progression [36]. The mRNA expression levels of proliferation-related genes (Survivin, PCNA, and Ki-67) were analyzed by qPCR following OLAP, CBD, and combination treatments.

**Fig. 4:**
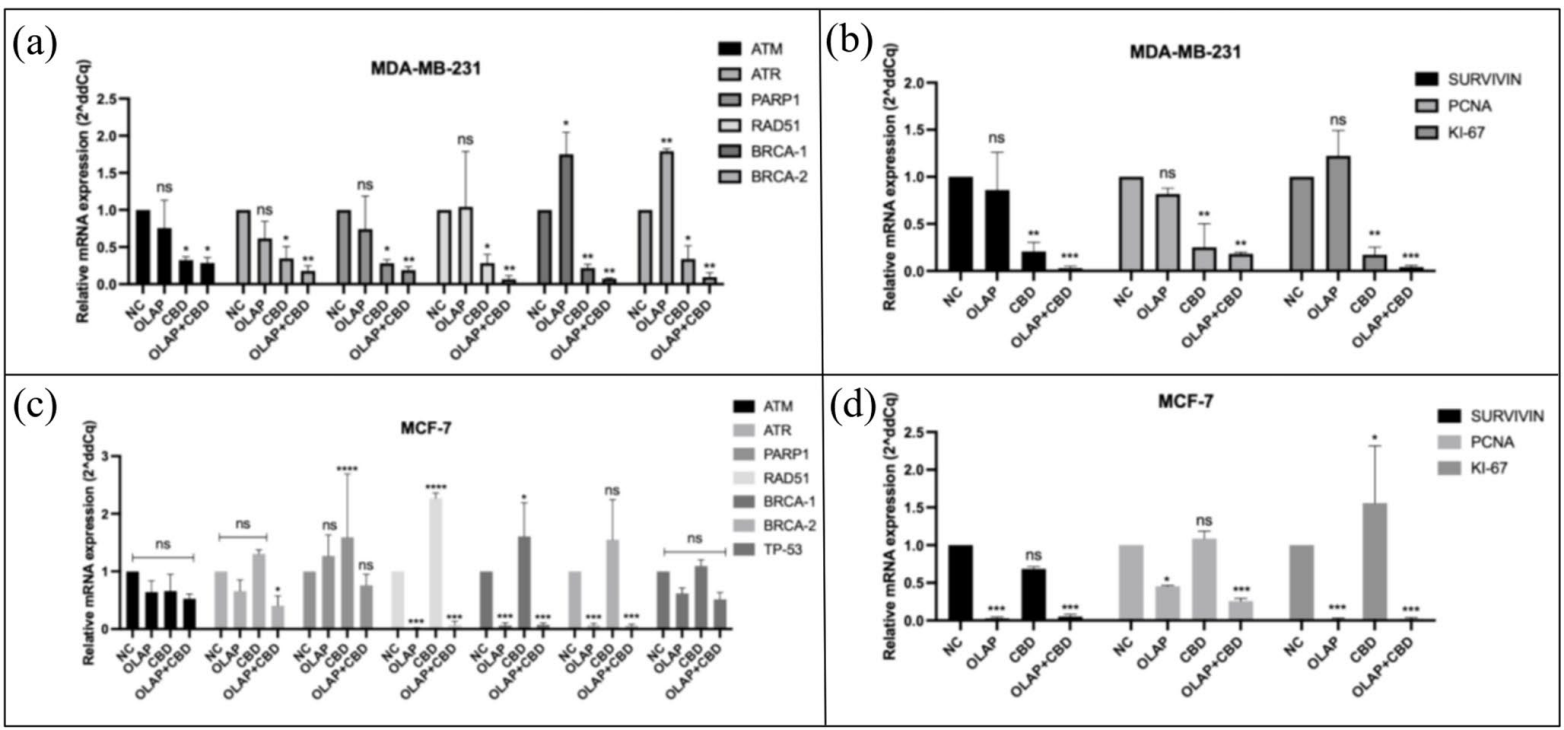
Effects of OLAP, CBD, and their combination on the expression of DNA damage and proliferation-related genes. Upper panels **a**, **b** show results for MDA-MB-231 cells, and lower panels **c**, **d** show results for MCF-7 cells. **a**, **c** Relative mRNA expression levels of DNA damage-related genes. **b**, **d** Relative mRNA expression levels of proliferation-related genes. All gene expression levels were determined by qPCR after 72 h of treatment. The abbreviation NC stands for negative control (untreated control cells). All data are presented as mean ± SD of three independent experiments (*n* = 3). Statistical significance is indicated as ns (non-significant), *p < 0.05, **p < 0.01, ***p < 0.001, ****p < 0.0001

In MDA-MB-231 cells, combination treatment resulted in a marked downregulation of DNA damage–related genes compared with the negative control (Fig. 4a). Relative to the control (set to 1.00), the expression levels of ATM, ATR, PARP1, RAD51, BRCA1, and BRCA2 were reduced to 0.28, 0.17, 0.18, 0.06, 0.07, and 0.09, respectively.

In MCF-7 cells, combination treatment also led to decreased expression of several DNA repair–related genes (Fig. 4c). Relative expression levels of ATR, RAD51, BRCA1, and BRCA2 were reduced to 0.40, 0.06, 0.07, and 0.057, respectively, compared with the negative control.

In MDA-MB-231 cells, combination treatment resulted in a marked downregulation of proliferation-related genes compared with the negative control (set to 1.00). Relative expression levels of Survivin, PCNA, and Ki-67 were reduced to 0.03, 0.18, and 0.04, respectively (Fig. 4b). Similarly, in MCF-7 cells, combination treatment led to decreased expression of the same proliferation markers. Relative expression levels of survivin, PCNA, and Ki-67 were 0.05, 0.25, and 0.022, respectively, compared with the negative control (Fig. 4d).

**Fig. 5:**
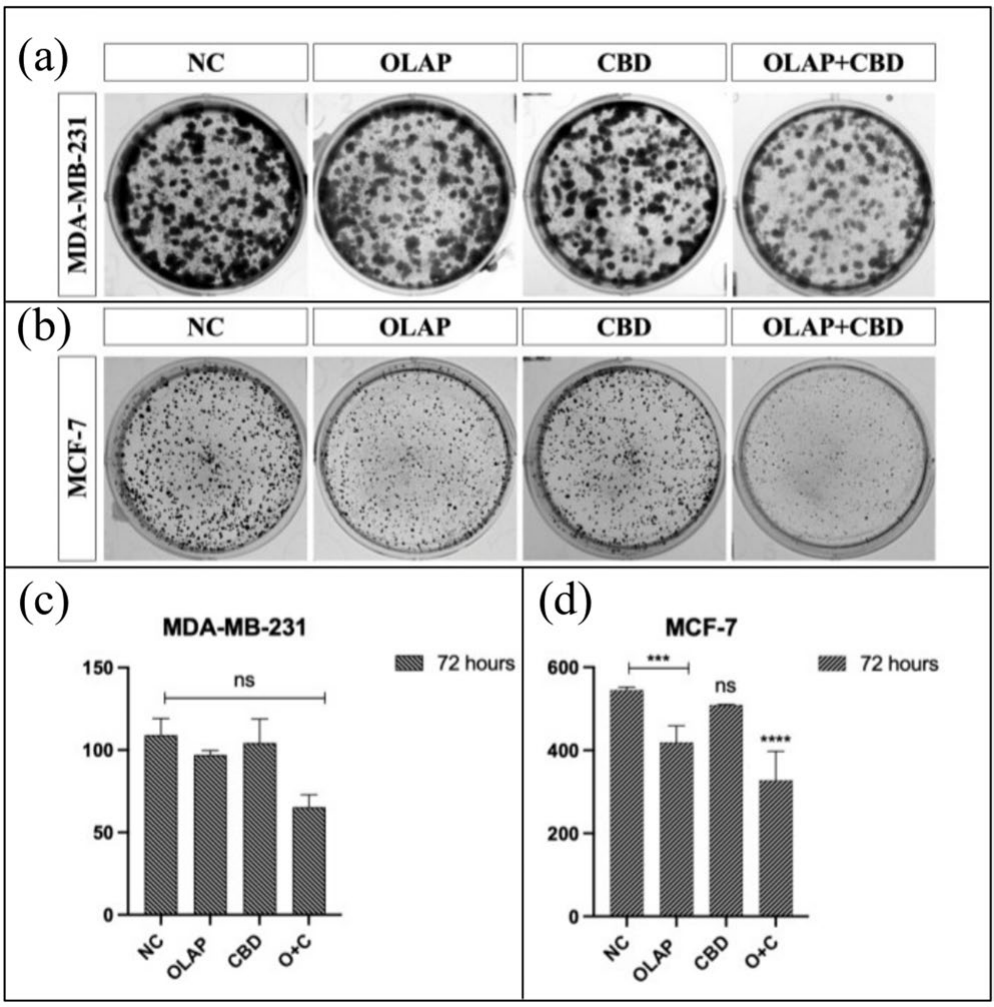
Effects of OLAP, CBD, and their combination on colony formation and cell migration in MDA-MB-231 and MCF-7 cells. **a**,** b** Representative images of colony formation and **c**,** d** quantitative analysis of colony numbers in both cell lines after 72 h treatment. The abbreviation NC stands for negative control (untreated control cells). All data are presented as mean ± SD of three independent experiments (*n* = 3). Statistical significance is indicated as ns (non-significant), *p < 0.05, **p < 0.01, ***p < 0.001, ****p < 0.0001

Colony formation and cell migration were assessed to evaluate the long-term proliferative and metastatic potential of OLAP, CBD, and their combination in MDA-MB-231 and MCF-7 cells. Colony formation analysis revealed a reduction in colony numbers in MCF-7 cells following combination treatment (Fig. 5b and d), whereas no significant change was observed in MDA-MB-231 cells compared with the negative control (Fig. 5a and c).

### Cell survival effects of olaparib, cannabidiol and their combination in 3D spheroid cultures of breast cancer cells

**Fig. 6:**
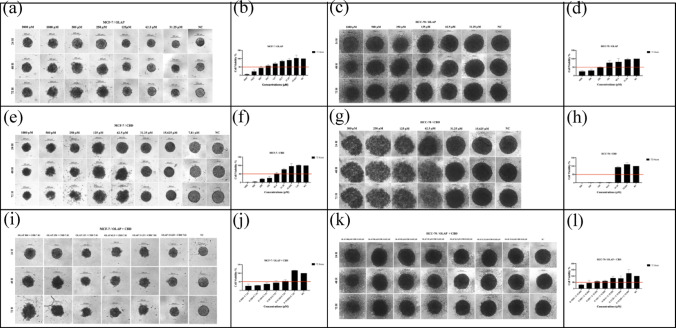
Comparative morphological and cytotoxic evaluation of 3D MCF-7 and HCC-70 spheroids. Panels **a**, **e**, **i** and **c**, **g**, **k** show representative images (captured at 24, 48, and 72 h) for MCF-7 and HCC-70, respectively. Corresponding 72 h dose-response curves (CellTiter-Glo^®^ 3D assay) are shown in panels **b**, **f**, **j** for MCF-7 and **d**, **h**, **l **for HCC-70. Rows represent treatments with **a**–**d** OLAP, **e**–**h** CBD, and **i**–**l** OLAP + CBD combination. **b**, **d** IC_50_ ​for OLAP was 250 µM in both lines. **f**, **h** IC_50_​ for CBD was 62.5 µM (MCF-7) and ≈ 50 µM (HCC-70). **j**, **l** Effective combination doses yielding 50% reduction in viability were 31.25 µM OLAP + 7.81 µM CBD for MCF-7, and ≈ 250 µM OLAP + 15.625 µM CBD for HCC-70. Data are presented as mean ± SD (*n* = 3). NC stands for negative control (untreated cells)

The cytotoxic effects of OLAP, CBD, and their combination were evaluated in 3D MCF-7 and HCC-70 spheroid models. Single-agent OLAP treatment induced dose and time dependent structural alterations. In MCF-7 spheroids, high concentrations resulted in significant shrinkage (Fig. 6a), whereas HCC-70 spheroids exhibited cellular dissociation and loss of spherical integrity (Fig. 6c). Despite these morphological differences, the 72 h IC_50_​ value for OLAP was 250 µM for both cell lines (Fig. 6b and d).

CBD treatment triggered a more rapid structural collapse in both models, which was clearly evident by 72 h (Fig. 6e, g). In the HCC-70 line, cellular dissociation was observed as early as 24 h at higher concentrations. The IC_50_ for CBD was determined to be approximately 62.5 µM in MCF-7 (Fig. 6f) and 50 µM in HCC-70 spheroids (Fig. 6h). The combination of OLAP and CBD resulted in pronounced structural disruption across the dose range in MCF-7 spheroids (Fig. 6i), with a markedly reduced effective concentration corresponding to a 50% reduction in viability (31.25 µM OLAP + 7.81 µM CBD) (Fig. 6j). In contrast, although structural dissociation was also observed in the HCC-70 spheroids (Fig. 6k), the effective concentration required to achieve 50% reduction in viability remained at 250 µM OLAP with 15.625 µM CBD (Fig. 6l), suggesting that CBD did not significantly reduce the effective dose range of OLAP in this 3D model.

## Discussion

Triple-negative breast cancer (TNBC) remains a clinically aggressive and therapy-resistant subtype, characterized by the lack of ER, PR, and HER2 expression and high rates of recurrence and metastasis [37]. While Olaparib has demonstrated therapeutic potential in tumors with genomic instability by targeting DNA damage repair, Cannabidiol (CBD) has been increasingly recognized for its antiproliferative and chemosensitizing effects [38, 39]. Building on these insights, the present study investigated the combined efficacy of Olaparib and CBD across different breast cancer models. Overall, the combination demonstrated enhanced antitumor activity in a cell line and model-dependent manner, supported by molecular and functional assays in both 2D and 3D systems. MTS-based viability analysis demonstrated that CBD increased the sensitivity of both MCF-7 and MDA-MB-231 cells to olaparib, resulting in reduced effective concentrations compared with monotherapy (Fig. 1). This is consistent with previous reports suggesting that CBD can modulate cellular stress responses and enhance susceptibility to DNA-damaging agents [38, 39]. It should be noted that IC_50_ values derived from metabolic assays primarily reflect changes in cellular metabolic activity rather than direct apoptotic induction. The concentrations used in functional assays were selected based on dose-response optimization and are in line with previously reported in vitro studies. Importantly, Combination Index (CI) analysis provided a quantitative assessment of drug interaction, revealing a modest synergistic effect in MDA-MB-231 cells (CI < 1), whereas an antagonistic interaction was observed in MCF-7 cells (CI > 1). These findings indicate that, although reduced viability was observed in both models, the combinational benefit is not uniform and is highly dependent on cellular context. Notably, this pattern is consistent with the limited apoptotic response and the absence of pronounced cell cycle arrest in MCF-7 cells. These differences may be attributed to intrinsic molecular characteristics of the cell lines, including BRCA-wild type status and caspase-3 deficiency, which are known to influence sensitivity to PARP inhibition and apoptotic signaling pathways.

Subtype-dependent differences were observed in downstream responses. In MDA-MB-231 cells, combination treatment induced apoptosis (Fig. 2a and b) and promoted G2/M associated cell-cycle arrest (Fig. 3a and b), accompanied by downregulation of key cell-cycle regulators (CDK1, CDK2, CDK4, and CDK6) (Fig. 3c), indicating disruption of proliferative signaling [40]. In contrast, MCF-7 cells did not show a strong apoptotic or phase-specific arrest response under similar treatment conditions (Figs. 2d and e and 3d and e). This finding is consistent with the known caspase-3 deficiency in MCF-7 cells, which limits classical executioner caspase-dependent apoptosis [41]. Therefore, in this context, observed transcriptional changes in cell-cycle regulators likely reflect a general reduction in proliferative capacity rather than discrete cell-cycle arrest detectable by flow cytometry (Fig. 3f) [42, 43].

Downregulation of DNA damage response-related genes following combination treatment may reflect sustained genotoxic stress induced by PARP inhibition and oxidative stress modulation by CBD. However, in the absence of protein-level validation of DNA damage signaling pathways, these findings should be interpreted as transcriptional indicators of cellular stress response rather than direct mechanistic confirmation. Similar adaptive transcriptional repression of repair-associated genes under prolonged stress conditions has been reported in the literature.

Caspase-3/7 activity assays confirmed increased apoptotic signaling in MDA-MB-231 cells following combination treatment (Fig. 2c). In contrast, MCF-7 cells exhibited minimal caspase activity changes, which is consistent with their caspase-3-deficient phenotype and suggests that alternative, caspase-independent mechanisms may contribute to growth inhibition in this model (Fig. 2f).

Colony formation assays further demonstrated differences in long-term proliferative capacity between cell lines. Although both cell lines exhibited comparable short-term cytotoxic responses, MCF-7 cells showed a more pronounced reduction in clonogenic survival following combination treatment, whereas MDA-MB-231 cells displayed only modest changes (Fig. 5) [43]. This apparent discrepancy between short-term antiproliferative effects (e.g., cell cycle arrest and gene expression changes) and the limited impact on long-term colony formation may reflect the use of sub-IC_50_ concentrations, which were selected to evaluate mechanistic responses while avoiding excessive cytotoxicity. Under these conditions, a subset of cells may retain the capacity to recover and sustain clonogenic growth over extended periods. This discrepancy may reflect intrinsic differences in clonogenic potential, survival signaling, and stress adaptation mechanisms between breast cancer subtypes [44, 45]. In particular, TNBC cells such as MDA-MB-231 are known to possess higher clonogenic resilience, which may contribute to their sustained long-term growth despite initial treatment-induced stress.

To bridge the gap between initial molecular findings and physiologically relevant conditions, 3D spheroid models were integrated into the study. While 3D architectures are traditionally associated with increased drug resistance due to cell–cell interactions, limited drug penetration, and metabolic gradients, the present results suggest that therapeutic response in complex cultures is highly context-dependent. The observed increased sensitivity in 3D models indicates that the combined effect of Olaparib and CBD may be influenced by microenvironmental stress conditions rather than being uniformly attenuated in spheroid systems. These findings highlight the importance of spheroid architecture, size, and metabolic state in shaping drug response, supporting the use of 3D models for more physiologically relevant evaluation of anticancer therapies [46].

The relatively small size of the spheroids generated in this study (≈ 250 μm) likely permitted adequate drug diffusion while simultaneously inducing hypoxic and metabolic stress, conditions known to impair DNA repair capacity and enhance the efficacy of PARP inhibition [47]. Due to the mesenchymal phenotype and low E-cadherin expression of MDA-MB-231 cells, which hindered the formation of compact and reproducible spheroids, HCC-70 cells were utilized as a technically robust TNBC model for 3D validation [48]. Importantly, HCC-70 was not used as a surrogate for MDA-MB-231, but as a stable representative of the TNBC subtype to ensure reproducible 3D architecture. Although differences in spheroid structure and assay platforms (MTS vs. CellTiter-Glo 3D) limit direct quantitative comparison of IC_50_ values, the overall trend supports enhanced treatment sensitivity in 3D conditions. This reinforces the relevance of the Olaparib and CBD combination in more physiologically relevant tumor models [49, 50].

Collectively, the results suggest that CBD may enhance the responsiveness of BRCA-wild type breast cancer cells to PARP inhibition, warranting further mechanistic investigation. The combined treatment showed consistent antiproliferative effects across models, although the magnitude of response varied depending on cellular context and culture dimensionality.

## Conclusion

In conclusion, the present study demonstrates that Olaparib and Cannabidiol (CBD), both as monotherapies and in combination, exert potent antiproliferative and cytotoxic effects across hormone receptor-positive (MCF-7) and triple-negative breast cancer (MDA-MB-231 and HCC-70) models in a context-dependent manner. The combinational response varied between models, with more pronounced effects observed in TNBC cells.

Notably, Combination Index (CI) analysis revealed that the interaction between OLAP and CBD is cell line–dependent, demonstrating a modest synergistic effect in MDA-MB-231 cells, whereas an antagonistic interaction was observed in MCF-7 cells (CI > 1). These findings underscore the critical role of molecular background in determining treatment response.

Importantly, 3D spheroid models further demonstrated that cellular architecture and microenvironmental conditions significantly influence drug sensitivity, highlighting the value of physiologically relevant systems in preclinical evaluation. The distinct response patterns observed between MCF-7 and TNBC models likely reflect underlying biological heterogeneity.

Although limited to in vitro systems and lacking protein-level validation of DNA damage signaling pathways, these findings provide supportive evidence for a context-dependent interaction between CBD and PARP inhibition. Future studies incorporating mechanistic validation and in vivo models will be essential to further elucidate the therapeutic potential of this combination in aggressive breast cancer.

Therefore, the therapeutic potential of OLAP and CBD co-treatment should be interpreted within a subtype-specific and molecularly defined framework.

## Electronic Supplementary Material

Below is the link to the electronic supplementary material.

Supplementary Material 1 Dose–response effects of cannabidiol (CBD), olaparib, and their combination in breast cancer cell lines.(a) Dose–response curve of CBD in MDA-MB-231 cells (IC₅₀ = 62.5 µM).(b) Dose–response curve of olaparib in MDA-MB-231 cells (IC₅₀ = 493.2 µM).(c) Dose–response curve of olaparib in combination with CBD in MDA-MB-231 cells (IC₅₀ = 78.93 µM).(d) Dose–response curve of CBD in MCF-7 cells (IC₅₀ = 48.8 µM).(e) Dose–response curve of olaparib in MCF-7 cells (IC₅₀ = 907.5 µM).(f) Dose–response curve of olaparib in combination with CBD in MCF-7 cells (IC₅₀ = 603.6 µM).Cells were treated with increasing concentrations of the indicated compounds for 72 h, and cell viability was assessed using the MTS assay. Data are presented as normalized absorbance (%) relative to the untreated control. Dose–response curves were generated using nonlinear regression analysis.

## Data Availability

No datasets were generated or analysed during the current study.
