# Supplementary figures and images for: Cannabidiol potentiates olaparib-induced cytotoxicity through cell cycle arrest and DNA damage modulation in breast cancer cells
Source: Mol Cell Biochem. 2026 Apr 28;481(6):2399–411. doi: 10.1007/s11010-026-05550-w (PMC13279729; doi:10.1007/s11010-026-05550-w)

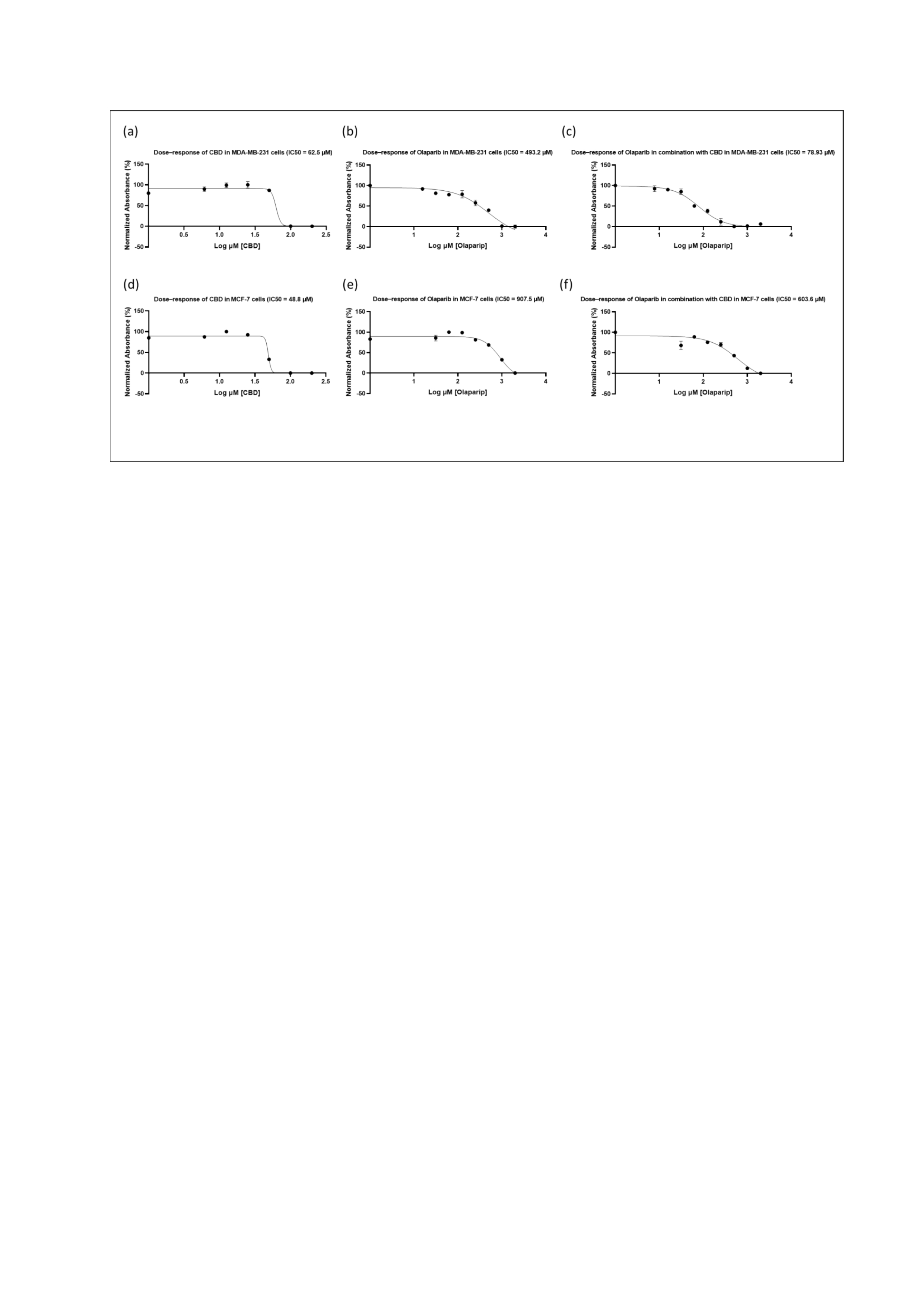

Supplement: Supplementary file 1 — Supplementary Material 1 Dose–response effects of cannabidiol (CBD), olaparib, and their combination in breast cancer cell lines.(a) Dose–response curve of CBD in MDA-MB-231 cells (IC₅₀ = 62.5 µM).(b) Dose–response curve of olaparib in MDA-MB-231 cells (IC₅₀ = 493.2 µM).(c) Dose–response curve of olaparib in combination with CBD in MDA-MB-231 cells (IC₅₀ = 78.93 µM).(d) Dose–response curve of CBD in MCF-7 cells (IC₅₀ = 48.8 µM).(e) Dose–response curve of olaparib in MCF-7 cells (IC₅₀ = 907.5 µM).(f) Dose–response curve of olaparib in combination with CBD in MCF-7 cells (IC₅₀ = 603.6 µM).Cells were treated with increasing concentrations of the indicated compounds for 72 h, and cell viability was assessed using the MTS assay. Data are presented as normalized absorbance (%) relative to the untreated control. Dose–response curves were generated using nonlinear regression analysis. [file 11010_2026_5550_MOESM1_ESM.jpg]
